# Development and Application of Fluorescent and Lateral Flow Dipstick Recombinase-Aided Amplification for Rapid Detection of *Glaesserella parasuis*

**DOI:** 10.3390/vetsci12080750

**Published:** 2025-08-12

**Authors:** Yongliang Che, Yao Wang, Renjie Wu, Longbai Wang, Xuemin Wu, Qiuyong Chen, Rujing Chen, Lunjiang Zhou

**Affiliations:** 1 Institute of Animal Husbandry and Veterinary Medicine, Fujian Academy of Agricultural Sciences, Fuzhou 350013, China; cyl19760810@163.com (Y.C.); wy1836507684@163.com (Y.W.); wurenjie1231@163.com (R.W.); wanglongbai@163.com (L.W.); wxm0593@163.com (X.W.); fjchenqiuyong@163.com (Q.C.); fjchenrujing@163.com (R.C.); 2 Fujian Animal Diseases Control Technology Development Center, Fuzhou 350013, China

**Keywords:** *Glaesserella parasuis*, recombinase-aided Amplification (RAA), development, application, diagnosis

## Abstract

*Glaesserella parasuis* (*G. parasuis*) is a bacterial respiratory pathogen from pigs, which can cause high morbidity and mortality in weaned piglets in piggery. An accurate and rapid detection for *G. parasuis* is crucial for the effective control of Glässer’s disease. In the present study, two distinct Recombinase-Aided Amplification (RAA) techniques, utilizing fluorescence and lateral flow dipstick, were developed for the detection of *G. parasuis*. The two RAA assays, namely the fluorescent RAA (Fluo-RAA) and lateral flow dipstick RAA (LFD-RAA) assays, prioritize specificity and are capable of detecting *G. parasuis* with sensitivities of 10 copies and 100 copies, respectively. Both assays are capable of utilizing boiled nasal swab samples as DNA templates, demonstrating sensitivity comparable to that of commercial DNA extraction kits. Sensitivity tests had revealed that the Fluo-RAA assay exhibited greater sensitivity than the LFD-RAA assay. While the Fluo-RAA assay is more sensitive than the LFD-RAA assay, it is also more labor-intensive and better-suited for laboratory use than for field conditions. The entire process for the LFD-RAA assay can be completed within one hour without the need for professional equipment, making it more suitable for field use.

## Simple Summary

Targeting the *rpoB* gene of *Glaesserella parasuis*, we developed the Recombinase-Aided Amplification with fluorescence (Fluo-RAA) or lateral flow dipstick (LFD-RAA) assays to detect *Glaesserella parasuis* in clinical samples. They are characterized by their rapidity, high sensitivity and specificity. Moreover, both assays have several advantages, such as simplified sample processing, rapid DNA amplification, and easy observation of results. The Fluo-RAA assay is more suitable for laboratory diagnosis, while the LFD-RAA assay is more suitable for field diagnosis.

## 1. Introduction

*Glaesserella parasuis* (*G. parasuis*), the etiological agent of Glässer’s disease, is one of the bacteria affecting pigs [1]. *G. parasuis*, an opportunistic pathogen, colonizes the upper respiratory tract of healthy and diseased pigs [2]. The Glässer’s disease caused by the bacterium is characterized by polyserositis, pneumonia, meningitis, and arthritis in porcine [1]. *G. parasuis* has 15 serotypes with different virulence, of which serotypes 4, 5, and 13 are more common serotypes, followed by serotype 12 and serotype 14 [3,4]. Also, the bacteria are mainly transmitted through the air, by direct contact, or via contaminated items. Weaned piglets are the most susceptible to *G. parasuis* infection. Co-infection with other bacteria is also common [5,6,7], which causes substantial economic losses in the pig industry worldwide [8].

Pneumonia and arthritis are two very common symptoms caused by some pathogenic bacteria, such as *G. parasuis*, *Actinobacillus pleuropneumoniae* (*A. pleuropneumoniae*) [9], *Pasteurella multocida* (*P. multocida*) [10], *Bordetella bronchiseptica* (*B. bronchiseptica*) [11], and *Actinobacillus indolicus* (*A. indolicus*) [12]. These bacteria often cause mixed infections in pig lungs, which are difficult to diagnose clinically. Thus, precise and rapid diagnosis for *G. parasuisis* is crucial for the prevention and control of Glässer’s disease. At present, diagnosis for the disease is only based on clinical features and pathological changes, and testing is less commonly performed. To enable actual detection, diagnostic methods are required. Several molecular detection methods, including PCR [13], real-time PCR [14], and loop-mediated isothermal amplification (LAMP) [15], have been developed for *G. parasuis* diagnosis. However, these technologies require expensive instruments and skilled professionals, which restricts their use in veterinary clinics [16]. Simpler and more convenient techniques are urgently required for the clinical diagnosis of *G. parasuis* in field conditions, which will offer guidance for the clinical use of targeted vaccines.

The Recombinase-Aided Amplification (RAA), the same as Recombinase Polymerase Amplification (RPA), is an isothermal amplification technology that can be used to detect pathogens with the advantages of rapidity, simplicity, and low cost, as seen in previous studies [17,18]. In the reaction, the recombinase (UvsX) pairs the specific primers with their homologous sequence in the genome, and the single-strand DNA binding protein (SSB) binds to the resulting displaced single-stranded DNA, preventing the immediate formation of double-stranded DNA [19]. Elongation is then achieved by the DNA polymerase, with the amplification being completed in 20–30 min at 39 °C [19]. Thus, the method is potentially very suitable for clinical application.

In this study, we aimed to develop a fluorescent RAA (Fluo-RAA) assay and a lateral flow dipstick RAA (LFD-RAA) assay for the diagnosis of *G. parasuis*. The analytical specificity and sensitivity of both assays were evaluated. Some clinical samples were collected to be tested, applying both assays.

## 2. Materials and Methods

### 2.1. Bacterial Strains and Clinical Samples

All bacterial strains used in this study, including *Glaesserella parasuis* (*G. parasuis*) (CVCC 3361), *Actinobacillus pleuropneumoniae* (*A. pleuropneumoniae*) (ATCC 43336), *Pasteurella multocida* (*P. multocida*) (CVCC 3048), *Bordetella bronchiseptica* (*B. bronchiseptica*) (ATCC 4617), and *Actinobacillus indolicus* (*A. indolicus*) strain 46KC2 (DSM 22264), were acquired from Beijing Baioubowei Biotech. Co., Ltd., Beijing, China, and preserved in our laboratory in 2023. Nasal swabs were collected from pigs in various swine farms in Fujian Province, China. These samples were collected by the authors of this study between July 2023 and July 2024. The details of these nasal swabs are listed in Table S1.

### 2.2. Bacterial Isolation and DNA Extraction

The nasal swabs collected were submerged in tryptic soy broth (TSB) (OXOID Ltd., Basingstoke, UK) and then mixed well. Subsequently, the mixture was plated on tryptic soy agar (TSA) (OXOID Ltd., Basingstoke, UK), supplemented with 0.005% nicotinamide adenine dinucleotide (NAD) (Amresco Inc., Solon, OH, USA), 5% defibrillated sheep blood (Zhengzhou Jiulong Biotech Co., Ltd., Zhengzhou City, China), and 5% bovine serum (Zhejiang Tianhang Biotechnology Co., Ltd., Hangzhou City, China). A single colony was obtained, and then the colony suspected of being *G. parasuis* was enriched and then stored for future use. The bacterial strain used in this study was resuscitated and inoculated into TSB medium to achieve the enrichment culture as previously described [20,21]. Bacterial genomic DNA was extracted using the Bacterial Genomic DNA Extraction Kit according to the manufacturer’s instructions (TianGen Biotech Co., Ltd., Beijing, China). A total of 121 pig nasal swab samples collected were treated, and the total DNA was extracted as previously described [22]. The extracted DNA was then stored at −20 °C for future use.

### 2.3. Primer and Probe Design

The specific primers and probes were designed for Fluo-RAA and LFD-RAA based on the *rpoB* gene sequence of *G. parasuis*, which was obtained from GenBank (https://www.ncbi.nlm.nih.gov/nucleotide/) (accession: CP001321.1) (accessed on 5 July 2024) and aligned using the multiple sequence alignment tool ClustalW [23]. The *rpoB* probe for Fluo-RAA was labeled with fluorescein FAM, while the *rpoB* reverse primer for LFD-RAA was labeled with 5′-biotin residues, Additionally, an internal probe for LFD-RAA was designed based on the RAA guidelines [17]. All oligonucleotide primers and probes were synthesized by Shangya Biotech Co., Ltd., located in Fuzhou City, China. These primers and probes are listed in Table S2.

### 2.4. Construction of Plasmid Standard

A partial sequence of the *rpoB* gene from *G. parasuis* was amplified by PCR using the following system: 2 × Taq Mix (Omega bio-tek, Norcross, GA, USA) of 25 µL, bacterial DNA template of 2 µL, forward (*rpoB*0-F) and reverse primers (*rpoB*0-R) (10 µM) of 1 µL each, distilled water of 21 µL filled to 50 µL. Reaction program: 94 °C for 5 min, denaturation at 94 °C for 45 s, annealing at 55 °C for 45 s, extension at 72 °C for 1 min, followed by 35 cycles of extension at 72 °C for 10 min, and storage at 4 °C. The target fragments were recovered by 1% agarose electrophoresis, purified, and connected to the pMD19-T vector (TaKaRa, Osaka, Japan), then transformed into competent cells DH5α (TaKaRa, Osaka, Japan). After the blue–white screening, suitable colonies were selected for overnight culture and plasmid extraction (Omega bio-tek, Norcross, GA, USA). The DNA copy number per unit volume of plasmid was calculated according to Moore’s law [24]. The calculation formula is as follows: plasmid copy number (copies/µL) = [plasmid concentration (g/µL) × 6.02 × 10^23^]/[total fragment length (bp) × 660 g/mol], total fragment length = vector length (bp) + fragment length (bp). The constructed standard plasmid was diluted to a gradient concentration of 10^5^–10^0^ copies/µL by a 10-fold dilution method and was stored at −20 °C for future use.

### 2.5. Development and Optimization for RAA Assays

Fluorescent RAA (Fluo-RAA) reactions were conducted at a constant temperature using Fluo-RAA kit (Hangzhou Zhongce Bio-Sci and Tech Co., Ltd., Hangzhou, China). The pre-mixed reagents comprised 25 μL of A buffer, 2.0 μL of each 10 μM RAA primer (*rpoB*1-F and *rpoB*1-R), 0.6 μL of a 10 μM probe (*rpoB*1-P), and 16.9 μL of distilled water. The pre-mixed reagents were added to the dry enzyme powder and mixed thoroughly. Subsequently, 1 μL of DNA template was introduced to the mixture, followed by the addition of 2.5 μL of B buffer into the lid of an open reaction tube. The reaction mixture was then centrifuged at 12,000 r/min for 10 s, after which the Fluo-RAA assay was conducted at a constant temperature. To determine the optimal reaction conditions, the RAA assays were conducted at temperatures ranging from 25 °C to 43 °C in a real-time PCR machine (BioRad CFX96 Touch, Pleasanton, CA, USA). The curve obtained from the Fluo-RAA assay depicted the fluorescence intensity, which positively correlates with the DNA yield on the *y*-axis, while the *x*-axis (abscissa) represents the cycle numbers of DNA amplification.

Lateral flow dipstick RAA (LFD-RAA) reactions were conducted using the LFD-RAA kit in a water bath (Hangzhou Zhongce Bio-Sci and Tech Co., Ltd., Hangzhou, China). The pre-mixed reagents comprised 25 µL of A buffer, 2.0 µL of each RAA primer (*rpoB*2L-F and *rpoB*2L-R) at 2 µM, 0.6 μL of a 2 μM probe (*rpoB*2L-P), and 16.9 μL of distilled water. The pre-mixed reagents were added to the dry enzyme powder and mixed thoroughly. Subsequently, 1 μL of DNA template was introduced to the mixture, followed by the addition of 2.5 μL of B buffer into the lid of an open reaction tube. Lastly, the reaction mixture was centrifuged at 12,000 r/min for 10 s, after which the RAA was conducted in a water bath. To achieve optimized reaction conditions, the RAA assay was conducted at the aforementioned temperatures in a water bath. The reaction products were diluted 1:6 in PBS as per the instructions provided in the LFD-RAA kit. In total, 100 μL of diluted reaction products was transferred to a new tube. Subsequently, dipsticks were inserted vertically into the tubes holding the diluted reaction mixtures. After allowing lateral flow dipsticks to incubate at room temperature for 15 min, the results were visually assessed.

### 2.6. Sensitivity and Specificity Test

The templates used in the sensitivity test for the Fluo-RAA assay were plasmid DNA, with a concentration ranging from 10^5^ to 10^0^ copies/μL, and the reaction results were observed through a fluorescence signal. Similarly, the templates used in the sensitivity test for the LFD-RAA assay were also plasmid DNA, with a concentration ranging from 10^5^ to 10^0^ copies/μL, and the reaction results were observed through the detection line of the dipsticks.

Using the optimized reaction system as a foundation, genomic DNA obtained from *G. parasuis*, *A. pleuropneumoniae*, *P. multocida*, *B. bronchiseptica*, and *A. indolicus* was, respectively, employed as the template, and primers and probes specific to the *rpoB* gene of *G. parasuis* were utilized to conduct specificity tests for both Fluo-RAA and LFD-RAA assays.

### 2.7. Sample Treatment Optimization

To develop a simple sample processing method that can be implemented in clinical practice, we investigated the boiling treatment method for nasal swabs and bacterial fluid samples. We prepared the nasal swabs solution by placing it in 2 mL of phosphate-buffered saline (PBS) and boiled it in a water bath for 5 min. Afterwards, the supernatant after centrifugation at 12,000 r/min for 5 min was used as the sample DNA. Meanwhile, the sample DNA was also extracted using a Bacterial Genomic DNA Extraction Kit. The DNA obtained through boiling methods and extraction kit methods could serve as a DNA template for Fluo-RAA and LFD-RAA assays.

### 2.8. Evaluation of the Clinical Samples

The samples collected from pigs were treated using commercial kits or boiling methods as described above. The DNA extracted or boiling supernatant was subjected to analysis by Fluo-RAA and LFD-RAA assays, respectively. The positive concordance rate of samples tested using Fluo-RAA and LFD-RAA assays was determined, and non-conforming samples were further confirmed by bacterial isolation and identification. The accuracy of both assays was evaluated.

## 3. Results

### 3.1. Development and Stability of Fluo-RAA and LFD-RAA Assays

**Figure 1:**
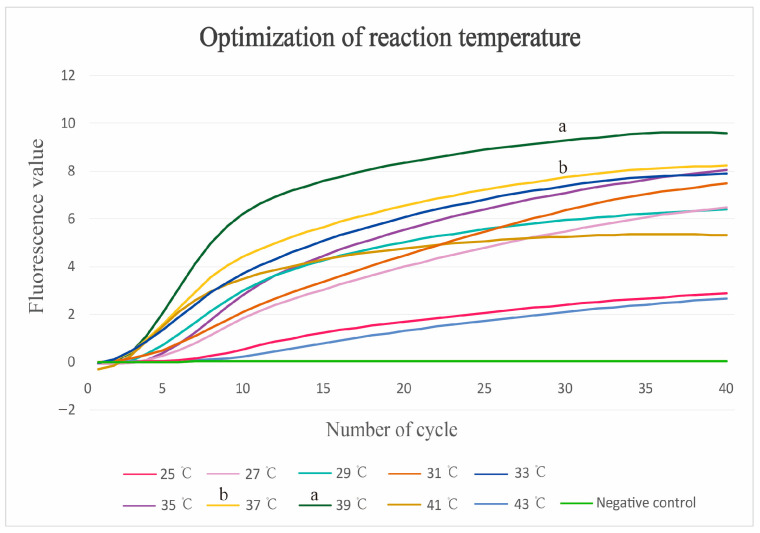
Optimization for Fluo-RAA assay. The Fluo-RAA assay is conducted at various temperatures. The fluorescence value of curve “a” is highest at 39 °C, whereas curve “b” comes second at 37 °C. The fluorescence values of all curves are lower, within the temperature ranges of 25–35 °C and 41–43 °C.

**Figure 2:**
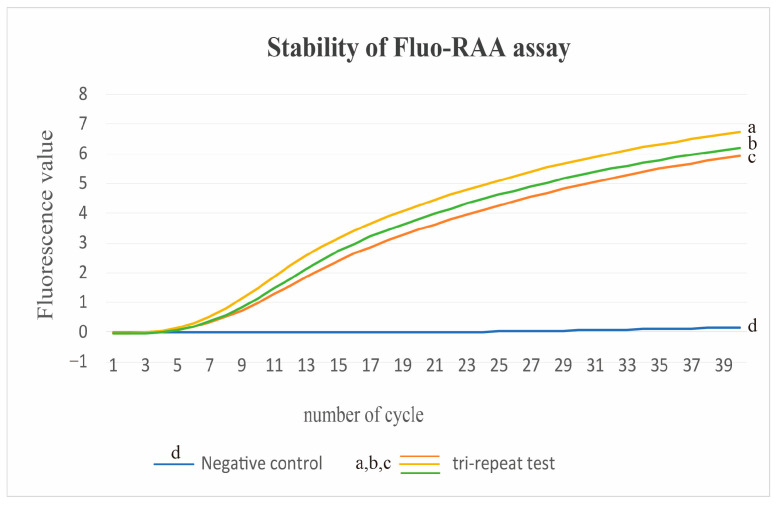
Stability test for Fluo-RAA assay. Three identical tests were carried out on the positive samples, yielding consistent reaction curves for a, b, and c, all derived from the same sample. Meanwhile, d represents the reaction curve of the negative control.

The RAA assay was conducted across a range of temperatures, from 25 °C to 43 °C, to determine the optimal reaction temperature. The fluorescence curve from the Fluo-RAA assay indicated that the DNA yield reached its peak at 39 °C (Figure 1). Based on these findings, the optimal reaction temperature for the RAA assays was determined to be 39 °C. Consequently, the LFD-RAA reaction temperature was also set at 39 °C. The stability of the Fluo-RAA assay was confirmed through three replicate tests on positive samples (Figure 2).

### 3.2. Sensitivity Test for Fluo-RAA and LFD-RAA Assays

**Figure 3:**
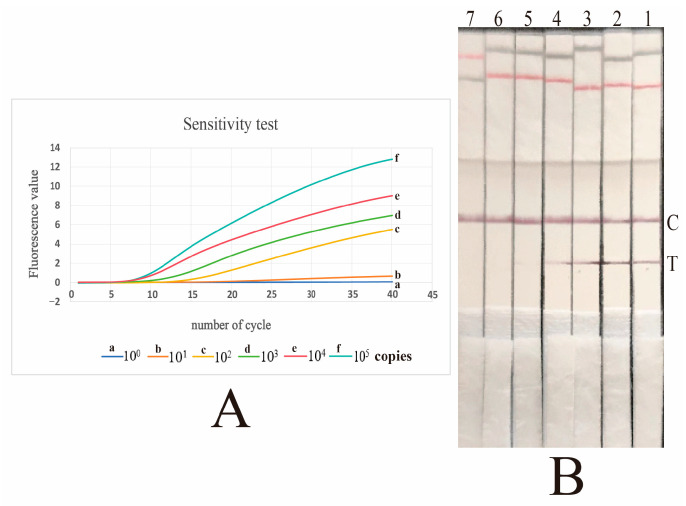
Sensitivity tests for Fluo-RAA and LFD-RAA assays. (**A**): The sensitivity test for the Fluo-RAA assay. The “a, b, c, d, e, and f” represent six distinct curves obtained by conducting the Fluo-RAA assay using various copies of DNA as the DNA template. The “a, b, c, d, e, and f” are 10^0^ copies, 10^1^ copies, 10^2^ copies, 10^3^ copies, 10^4^ copies, and 10^5^ copies, respectively. The sample from curve “a” is detected as negative by the Fluo-RAA assay, whereas all the samples from curves “b, c, d, e, f” are detected as positive by the same assay. (**B**): The sensitivity tests for the LFD-RAA assay. “C” represents the control line, and “T” denotes the detection line. The seven dipsticks labeled “1, 2, 3, 4, 5, and 6” correspond to the detection results obtained from various DNA template copies, with the number of DNA copies being 10^5^ copies, 10^4^ copies, 10^3^ copies, 10^2^ copies, 10^1^ copies, and 10^0^ copies, respectively. Additionally, “7” denotes the detection outcome from distilled water. The four dipsticks labeled “1, 2, 3, and 4” exhibit a positive result, whereas the three dipsticks labeled “5, 6, and 7” show a negative result.

The RAA assays were performed using serially diluted standard plasmids (10^5^–10^0^ copies per reaction) as a DNA template. The results showed that template DNA could only be detected as positive by the Fluo-RAA assay when its concentration reached 10 or more copies per reaction. However, the LFD-RAA assay could detect template DNA down to a minimum of 100 copies. In other words, the sensitivities of the Fluo-RAA assay and the LFD-RAA assay are 10 copies and 100 copies of template DNA, respectively, as shown in Figure 3.

### 3.3. Specificity Test for Fluo-RAA and LFD-RAA Assays

**Figure 4:**
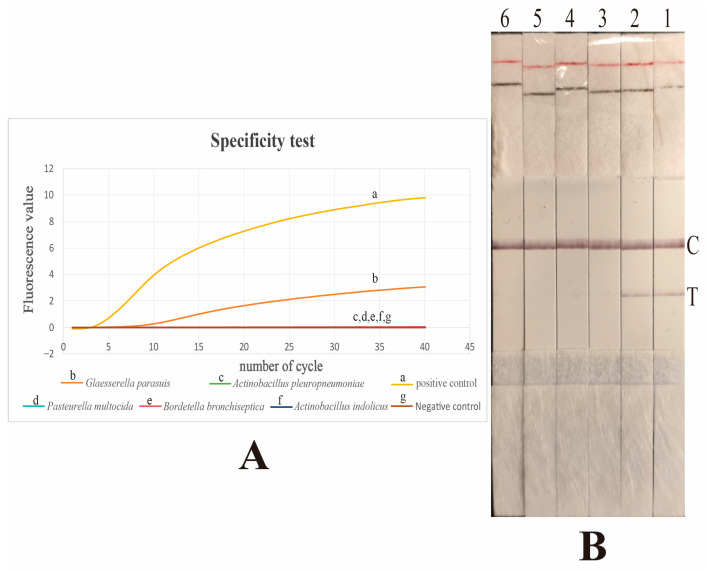
The specificity tests for the Fluo-RAA and LFD-RAA assays. (**A**): The specificity test for the Fluo-RAA assay. The “a, b, c, d, e, and f” represent six distinct curves obtained by conducting the Fluo-RAA assay using various copies of DNA as the DNA template. The curves labeled “a, b, c, d, e, f, and g” represent distinct curves obtained through the conducted Fluo-RAA assay using various DNA templates. The DNA templates are derived from positive plasmids, *Glaesserella parasuis*, *Actinobacillus pleuropneumoniae*, *Pasteurella multocida*, *Bordetella bronchiseptica*, and *Actinobacillus indolicus*, and a negative control, respectively. Out of these, only the curves from positive plasmids and *Glaesserella parasuis* exhibit positive results, while all other curves are negative. (**B**): The specificity test for the LFD-RAA assay. The dipsticks labeled “1, 2, 3, 4, 5, and 6” represent the detection result obtained through the conducted LFD-RAA assay using various DNA templates. The DNA templates are derived from positive plasmids, *Glaesserella parasuis*, *Actinobacillus pleuropneumoniae*, *Pasteurella multocida*, *Bordetella bronchiseptica*, and *Actinobacillus indolicus*, respectively. Only the detection results obtained from positive plasmids and *Glaesserella parasuis* are positive, whereas all other detection results are negative. In this context, “C” denotes the control line, while “T” signifies the detection line.

The specificity of the Fluo-RAA and LFD-RAA assays was evaluated for their ability to differentiate between *G. parasuis* and other related bacterial pathogens from pigs. The results showed that both Fluo-RAA and LFD-RAA assays were only capable of detecting *G. parasuis* and no other bacteria. In conclusion, both the Fluo-RAA assay and LFD-RAA assay demonstrated specificity in detecting *G. parasuis*. The results are presented in Figure 4.

### 3.4. Simplified Procedure and Clinical Evaluation for Fluo-RAA and LFD-RAA Assays

To ensure that the Fluo-RAA and LFD-RAA assays are suitable for clinical diagnosis, a simplified DNA extraction procedure was tested. DNA was extracted from nasal swab samples containing *G. parasuis* using commercial kits or boiling treatment, and then the Fluo-RAA and LFD-RAA assays were performed. The results showed that the outcomes for the Fluo-RAA and LFD-RAA assays using sample DNA obtained through boiling treatment as the DNA template were consistent with those using sample DNA extracted using commercial kits as the DNA template. Therefore, the results indicated that boiling the samples was a feasible alternative method to DNA extraction in Fluo-RAA and LFD-RAA assays.

**Figure 5:**
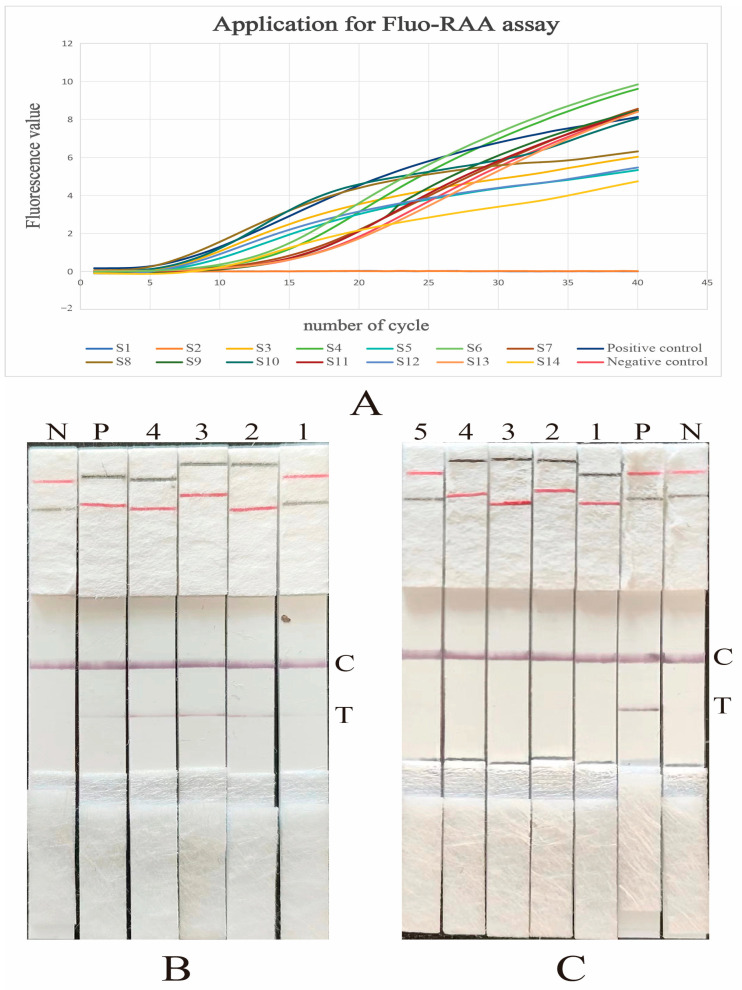
Application test for Fluo-RAA and LFD-RAA assays. “N” denotes negative control, “P” signifies positive control, and “C” indicates control lines for LFD quality assurance. (**A**): Application test for the Fluo-RAA assay. The “S1–S14” represent 14 distinct samples. (**B**): Application test for positive samples in the LFD-RAA assay; “1–4” represent 4 distinct positive samples. (**C**): Application test for negative samples in the LFD-RAA assay; “1–5” represent 5 distinct negative samples.

A total of 121 nasal swab samples and 48 bacterial strains were collected from pigs suffering from respiratory diseases. These samples were then tested for *G. parasuis* using both Fluo-RAA and LFD-RAA assays. Out of these nasal swab samples, 26 tested positive for *G. parasuis* by the Fluo-RAA assay, while 24 tested positive for *G. parasuis* using the LFD-RAA assay. The positive rates for the two assays were 21.5% and 19.8%, respectively. Furthermore, among these bacterial strains, 12 tested positive in both the Fluo-RAA and LFD-RAA assays, with a positive rate of 25%. Specifically, two samples that were initially identified as positive for *G. parasuis* by the Fluo-RAA assay but negative by the LFD-RAA assay were subsequently confirmed as positive for *G. parasuis* through bacterial isolation and 16S rRNA sequencing [25]. The discrepancy in the LFD-RAA results for the two samples was attributed to the presence of fewer *G. parasuis* copies. It is noteworthy that all samples underwent boiling treatment to obtain DNA templates, and the results are presented in Table 1 and Figure 5.

## 4. Discussion

As an opportunistic pathogen, *G. parasuis* infection can lead to disease when immunity decreases in pigs. The pathogenic bacterium is usually regarded as a secondary pathogen or a co-infectious pathogen, which often co-infects pigs with other related pathogenic pathogens [26,27]. Thus, to offer a scientific basis for the prevention and control of Glässer’s disease, it is imperative to develop a detection method that can detect *G. parasuis* with high speed, sensitivity, and specificity.

Due to its rapidity, sensitivity, specificity, and simplicity, molecular diagnostic techniques have been widely applied for detecting various microorganisms and specific genes. However, the requirement for specialized instruments restricts the field use of certain methods, such as PCR [13], real-time PCR [14], LAMP [15], and Fluo-RAA [18,19]. The LFD-RAA [17] method has advantages over PCR, real-time PCR, LAMP, and Fluo-RAA in terms of convenience and equipment requirements and has been developed and applied for pathogenic detection in field conditions.

During the reaction of the RAA assay, the compound, formed by the recombinase and the primers, identifies the homologous sequence, followed by a chain replacement reaction. This reaction can rapidly amplify new DNA fragments in vitro after a 15–30 min reaction under isothermal conditions [28]. In this study, the LFD-RAA assay combined RAA with LFD, utilizing an nfo probe (46 bp) labeled with fluorescein (FAM) and reverse primer (35 bp) labeled with biotin to realize the visualization of the detection results. The probe has a tetrahydrofuran (THF) at least 30 bases away from the 5′-end and at least 15 bases away from the 3′-end, and the 3′-end is modified with a blocking group (C3-spacer). The endonuclease does not work when the probe is single-stranded, but it can recognize and cleave THF when the probe binds to the homologous sequence to form a double-stranded amplification product, and finally forms a double-labeled amplification product with FAM and biotin. The detection results can be obtained within 3 min through LFD, realizing rapid and visual field detection. The Fluo-RAA assay combines RAA with an exo probe (43 bp), which greatly improves the detection sensitivity and enables the detection of low-copy samples. Similarly, the THF site is labeled in the middle of the probe and required to be at least 30 bases away from the 5-end and 15 bases away from the 3′-end. A fluorescent group (FAM) and a quenching group (BHQ1) were marked on both sides of the THF site, respectively, and a blocking group (C3-spacer) was modified at the 3′-end. When the probe is single-stranded, the exonuclease does not work, and the fluorescent signal emitted by the fluorescent group is absorbed by the quenching group. When the probe is combined with the homologous sequence to form a double-stranded amplification product, the exonuclease can recognize and cut THF so that the signal emitted by the fluorescent group can be collected. With the extension of the amplification time, the fluorescent signal is continuously accumulated, which can be monitored in real-time by the corresponding instrument.

Based on the aforementioned principle, in this study, we have developed Fluo-RAA and LFD-RAA assays for the detection of *G. parasuis* infection using primers and probes that target the *rpoB* gene. The *rpoB* gene codes for bacterial RNA polymerase and displays remarkable conservation in both structure and sequence [29]. Thus, the *rpoB* gene can serve as a target gene for the development of an RAA assay. The sensitivity and specificity of the methods were determined. The minimum detectable amount for the Fluo-RAA assay was 10 copies, whereas the minimum detectable amount for LFD-RAA was 100 copies. In comparison, the Fluo-RAA assay exhibited higher sensitivity than the LFD-RAA assay. Through the application of the two assays to the test of boiling nasal swab samples, it was further confirmed that the Fluo-RAA assay exhibits higher sensitivity. The specificity test indicated that both Fluo-RAA and LFD-RAA assays could only detect *G. parasuis*, which suggested that both assays demonstrated good specificity.

The Fluo-RAA assay was conducted within a reaction temperature range of 25–43 °C. The fluorescence curve obtained by the Fluo-RAA assay showed that the DNA yield was highest at 39 °C, which was consistent with previous studies [30,31]. However, the standard plasmids all tested positive for *G. parasuis* at 25–43 °C. Therefore, for RAA assays that do not require a specific reaction temperature, the LFD-RAA assay is the optimal choice for detecting clinical samples in field conditions [32,33].

To simplify DNA template preparation for the RAA assay, we employed a boiling treatment procedure. Bacteria were collected and spiked into PBS, and the supernatant was then used as a DNA template for the RAA assay. The results revealed that the boiling treatment had equivalent sensitivity to the kit treatment. The simplified sample treatment procedure is highly valuable for field use due to its high sensitivity. Boiling treatment eliminates the need for specific equipment, enabling the LFD-RAA assay to be conducted on farm or other field environments.

Finally, the Fluo-RAA and LFD-RAA assays were applied to assess the clinical samples. The detection positive rate for the Fluo-RAA assay was 21.5% (26/121), whereas for the LFD-RAA assay, it was 19.8% (24/121). All 24 positive samples identified by the LFD-RAA assay were also confirmed positive by the Fluo-RAA assay. Through bacterial isolation, two samples with inconsistent results were also confirmed positive for *G. parasuis*. Therefore, the results indicated that the sensitivity of the Fluo-RAA assay was higher than that of the LFD-RAA assay, which was consistent with the sensitivity test of both RAA assays. This finding suggested that both the Fluo-RAA and LFD-RAA assays performed well in detecting clinical samples and possessed potential for application in the diagnosis of *G. parasuis*. While the Fluo-RAA assay is better-suited for laboratory diagnosis, the LFD-RAA assay is more appropriate for field conditions. Additionally, both Fluo-RAA and LFD-RAA assays can be conducted in less than an hour without requiring specialized equipment or DNA extraction steps, thereby significantly increasing detection speed and expanding their range of applications.

In conclusion, by targeting the *rpoB* gene, we developed Fluo-RAA and LFD-RAA assays to detect *G. parasuis* in clinical samples. These assays are characterized by their rapidity, high sensitivity, and specificity. Additionally, both assays have several advantages, such as simplified sample processing, rapid DNA amplification, and easy observation of results. The Fluo-RAA assay is more suitable for laboratory diagnosis, while the LFD-RAA assay is more appropriate for field diagnosis.

## Figures and Tables

**Table 1:** Clinical application of Fluo-RAA and LFD-RAA assays.

Number of Samples		Nasal Swabs	Bacterial Strains
Number of samples		121	48
Positive number by Fluo-RAA	DNA extraction kit	26	12
	Boiling treatment	26	12
Positive number by LFD-RAA	DNA extraction kit	24	12
	Boiling treatment	24	12

Note: “DNA extraction kit” refers to template DNA extracted by the DNA extraction kit. “Boiling treatment” refers to template DNA by boiling treatment.

## Data Availability

All data generated or analyzed during this study are available from the corresponding author upon reasonable request.

## Supplementary Materials

The following supporting information can be downloaded at https://www.mdpi.com/article/10.3390/vetsci12080750/s1. Table S1: The information of collection samples; Table S2: All primers and probes used in this study.
