# Supplementary material for: Development and Application of Fluorescent and Lateral Flow Dipstick Recombinase-Aided Amplification for Rapid Detection of Glaesserella parasuis
Source: Vet Sci. 2025 Aug 12;12(8):750. doi: 10.3390/vetsci12080750 (PMC12389767; doi:10.3390/vetsci12080750)
Supplement: Supplementary file 1 [file vetsci-12-00750-s001.zip › Table S1.pdf]

Table S1. The information of collection samples

No.	Samples source (number of samples)	Collection time	Collection location	Pig age	Healthy status	Bacterial strains
1	Nasal swabs of pigs (11)	05/08/2023	Sanming City	35-70 d	Both ill and heathy	4
2	Nasal swabs of pigs (15)	29/07/2023	Zhangzhou City	35-70 d	Both ill and heathy	7
3	Nasal swabs of pigs (16)	19/03/2024	Nanping City	35-70 d	Both ill and heathy	7
4	Nasal swabs of pigs (18)	06/11/2023	Longyan City	35-70 d	Both ill and heathy	8
5	Nasal swabs of pigs (18)	10/09/2023	Zhangzhou City	35-70 d	Both ill and heathy	6
6	Nasal swabs of pigs (21)	03/04/2023	Ningde City	35-70 d	Both ill and heathy	8
7	Nasal swabs of pigs (22)	17/05/2024	Quanzhou City	35-70 d	Both ill and heathy	8
