# Supplementary material for: Development and Application of Fluorescent and Lateral Flow Dipstick Recombinase-Aided Amplification for Rapid Detection of Glaesserella parasuis
Source: Vet Sci. 2025 Aug 12;12(8):750. doi: 10.3390/vetsci12080750 (PMC12389767; doi:10.3390/vetsci12080750)
Supplement: Supplementary file 1 [file vetsci-12-00750-s001.zip › Table S2.pdf]

**Table S2.** All primers and probes used in this study

| No. | Names | Sequence (5'→3') | Target fragment |
|---|---|---|---|
| 1 | rpoB0-F | TCGTGTACCAAACGGCACAT | 465 bp |
| | rpoB0-R | TTGTTACCGTGACGACCTGC | |
| 2 | rpoB1-F | GTGAATGCCAAATTCGTGGTACAACCTATGCAGC | 170 bp |
| | rpoB1-R | GTTGATCACGAAAGTACCGTTGTCGGTCATCAATG | |
| | rpoB1-P | CCTTTACGCGTAAAACTTCGTCTTGTGACC(FAM)(THF)(BHQ1)GATCGCGAAGCG(C3-spacer) | |
| 3 | rpoB2L-F | GTGAATGCCAAATTCGTGGTACAACCTATGCAGC | 170 bp |
| | rpoB2L-R | (Biotic)GTTGATCACGAAAGTACCGTTGTCGGTCATCAATG | |
| | rpoB2L-P | (FAM)CCTTTACGCGTAAAACTTCGTCTTGTGACCTT(THF)TGATCGCGAAGCG(C3-spacer) | |

Note: "-F" represents forward primer, "-R" represent reverse primer, "-P" represent probe. "FAM" is a fluorescent group, "BHQ1" is a quenching group, "THF" is tetrahydrofuran, "C3-spacer" is a blocking group, "Biotic" is a label group.
